# Condylectomy and “surgery first” approach: An expedited treatment for condylar hyperplasia in a patient with facial asymmetry

**DOI:** 10.1590/2177-6709.22.4.086-096.oar

**Published:** 2017

**Authors:** Diego Fernando López, Juan Fernando Aristizábal, Rosana Martínez-Smit

**Affiliations:** 1 Universidad del Valle, Facultad de Salud, Escuela de Odontología, Departamento de Ortodoncia (Cali/ Valle del Cauca, Colombia).; 2 Universidad CES, Facultad de Odontología, Departamento de Ortodoncia (Medellín/Antioquia, Colombia).

**Keywords:** Condylar hyperplasia, Surgery first, Orthodontics.

## Abstract

Condylar Hyperplasia (CH) is a self-limiting pathology condition that produces severe facial deformity at the expense of mandibular asymmetry. In this case report a 15-year-old female patient was diagnosed with Unilateral Condylar Hiperplasia (UCH) by mean of single-photon emission computed tomography (SPECT) and histological study. A high condylectomy in the right condyle was performed to stop the active status of the hyperplasia. A month after condylectomy, orthognathic jaw impaction and asymmetric mandibular setback surgery was performed with the Surgery First Approach (SFA). After 10 days, orthodontic appointments were made every two weeks during 4 months. The active phase of treatment lasted 14 months. Excellent facial and occlusal outcomes were obtained and after 24 months in retention the results remained stable.

## RESUMO

A hiperplasia condilar (HC) é uma condição patológica autolimitante que produz deformidades faciais severas devido à assimetria mandibular. Nesse estudo de caso, uma paciente de 15 anos de idade foi diagnosticada com hiperplasia condilar unilateral (HCU), por meio de uma tomografia computadorizada por emissão de fóton único (SPECT) e de um estudo histológico. Uma condilectomia alta no côndilo direito foi realizada para interromper o *status* ativo da hiperplasia. Um mês após o procedimento, foram realizadas a impacção ortognática de maxilar e a cirurgia de retroposicionamento assimétrico da mandíbula, por meio de abordagem do tipo benefício antecipado. Depois de 10 dias, consultas ortodônticas passaram a ser feitas a cada duas semanas, durante 4 meses. A fase ativa do tratamento durou 14 meses. Excelentes resultados oclusais e faciais foram obtidos e, após 24 meses de contenção, os resultados permaneceram estáveis.

## INTRODUCTION

Condylar Hyperplasia (CH) is a self-limiting pathology condition that produces severe facial deformity at the expense of mandibular asymmetry^1^. It is commonly found in patients aged 11 to 25 years in its active form.^2^ It is characterized by exaggerated and progressive growth of the condyle’s head and neck, the ramus and the body of the mandible. Affects the facial esthetics, masticatory function and the joint health. It has been reported to be more prevalent in women than in men.^3^ ^,^ ^4^

The CH etiology is due to factors that may be genetic, traumatic, functional, hormonal and tumor-related.^5^ ^,^ ^6^ The diagnosis of CH should be performed by a combination of clinical and radiological findings and then corroborated by means of medicine nuclear test such as scintigraphy^1, 4^ and bone scan SPECT (Single-photon emission computed tomography) which is a sensitive and accurate method of detecting the growth activity of this disorder. This method can be used to quantitate the radionuclide uptake differences between the left and right condyles. Uptake differences of 10% or more between the left and right condyles, with increased uptake ipsilateral to the CH, are considered to be evidence of active growing CH. Quantitative assessment of CH is important to select an appropriate treatment course. Degenerative arthropathies of the temporomandibular joints may result in altered uptake, but this is mostly associated with the side contralateral to the CH. The CT portion of SPECT/CT is useful to assess the condylar dimensions and underlying bony changes. Bone scan SPECT also has demonstrated a superior performance in this application to planar imaging through the accurate and quantifiable assessment of mandibular condylar growth.^7^ ^-^ ^9^ The CT portion of SPECT/CT provides detailed anatomic information associated with growth activity. 

Early diagnosis is important since treatment modalities considerably differ depending on the affected structures, patient’s age, severity of the asymmetry and the active or passive status of the pathology.^1^ ^,^ ^4^

In many cases when the pathology is active, besides removing the growth site in the condyle, is necessary to perform orthognatic surgery to correct the deformity.^10^ Usually in the first place the condylar surgery is performed, then orthodontic treatment and finally orthognatic surgery.^11^ ^,^ ^12^ But recently the “Surgery First Approach” (SFA) might be an option. 

SFA recently introduced avoids deterioration of aesthetics and function especially in cases of skeletal Class III due to the absence of pre-surgical orthodontics^13^ ^-^ ^17^. Also, patients can benefit from immediate improvement of facial profile after surgery and shorter treatment time.^13^ ^-^ ^17^ Reduction of treatment time is possible because the regional acceleration phenomenon (RAP)^18^ and the dental decompensation is partially solved by the surgery.^19^

RAP is a complex physiological process with specific characteristics that includes rapid bone remodeling and loss of regional bone density. RAP increases tissue reorganization and healing by means of transitory increase of localized bone resorption and further remodeling.^20^ ^,^ ^21^ This is the explanation of the acceleration of the tooth movement for a period of 4 to 5 months approximately. 

The purpose of this report is to present a case of a Class III asymmetric patient with condylar hyperplasia in which SFA was used combined with condylectomy. 

## DIAGNOSIS AND ETIOLOGY

**Figure 1:**
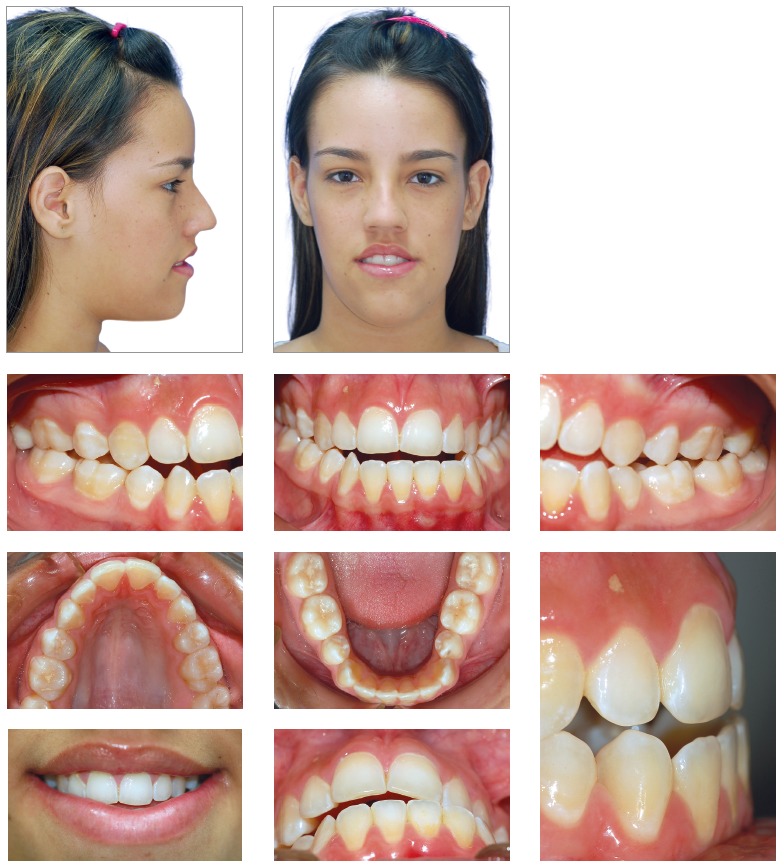
Pretreatment photographs.

A 15-year-old female patient, no medical background, previous orthodontic treatment with premolar extractions, looking for retreatment, with the following chief complaint: *“After the orthodontic treatment my face is deviated and the occlusion is very unstable”*. During the functional analysis the patient was diagnosed with tongue thrust, mandibular deflection to the left side and lip incompetence. The patient had a concave profile, increased lower facial third, short upper lip, commissure asymmetry with maxillary and occlusal planes canted (Fig 1 and Table 1).

**Table 1:** Cephalometric measurements: Initial cephalogram (T_1_) and final cephalogram (T_2_).

Measurement	T_1_	T_2_
SNA	77^o^	76^o^
SNB	78^o^	75^o^
ANB	-1^o^	1^o^
WITS	-5 mm	-1 mm
U1 - PP	110º	111^o^
IMPA	84º	89^o^
S-N/ Go-Gn	41º	33^o^
Ar-Go-Me	137º	129^o^
ANS-Me	81 mm	71 mm
Co-Gn	129 mm	122 mm
Na-Me/ S-Go	59.3%	62%

**Figure 2:**
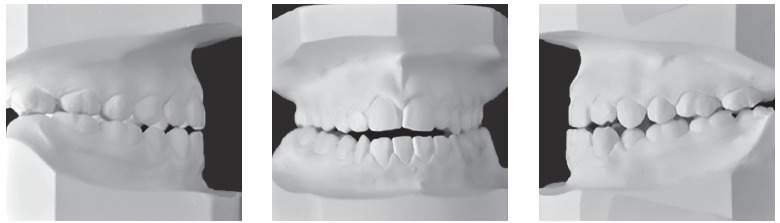
Pretreatment dental casts.

The patient presented a Class III malocclusion, posterior crossbite, anterior open bite, narrow arches and lower midline deviated to the left side (Figs 1 and 2).

**Figure 3:**
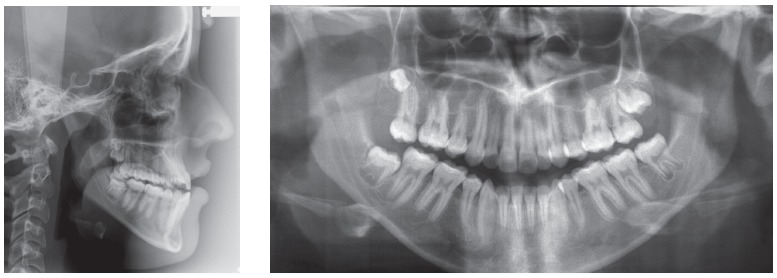
Pretreatment radiographs.

Skeletally she presented a Class III hyperdivergent pattern, dolichofacial biotype and severe retroclined lower incisor; long, thin and asymmetrical condyles (Fig 3).

**Figure 4:**
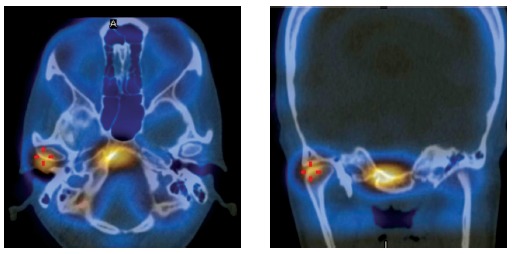
Single-photon emission computed tomography showing abnormal uptake in the right condyle.

The SPECT examination showed area of abnormal captation (62%) in the right condyle (Fig 4), which confirmed the suggestive chondroblast hyperactivity of the unilateral condylar hyperplasia (UCH).^22^

## TREATMENT OBJECTIVES

Both the skeletal and dental objectives of the treatment plan were closely related, which included: 1) To stop condylar hyperplasia, 2) To fix skeletal discrepancy, 3) To correct the malocclusion and aesthetic requirements of the patient.

## TREATMENT ALTERNATIVES

These were the treatment options: 1) pre-surgical orthodontic treatment, followed by maxillofacial and condylar surgery; 2) condylectomy and “surgery-first approach”, followed by orthodontics to align, level and to stabilize the occlusion, and 3) condylectomy and orthodontic dental compensation of the malocclusion.

Having in mind that the patient’s chief concern was her facial esthetics, it was decided to go on with surgical treatment. SFA was chosen because the patient wanted immediate facial change, avoiding deterioration of her profile and malocclusion, and also taking advantage of all the biological potential of regional acceleration. 

## TREATMENT PROGRESS

A high condylectomy in the right condyle was performed to stop the active status of the hyperplasia, removing 5 mm of height from medial pole to lateral pole. The specimen was sent to pathology and histological report showed significant extensions of cartilage that exceeded the osteochondral junction and increase in the thickness of the layers of the condyle.

One day before surgery, interactive self-ligating brackets (In-Ovation R CCO^®^, 0.022 x 0.028-in slot, GAC International Inc., Bohemia, USA) were bonded, and thermoelastic 0.014-in Sentalloy arches (Dentsply GAC) were placed before starting surgery. 

**Figure 5:**
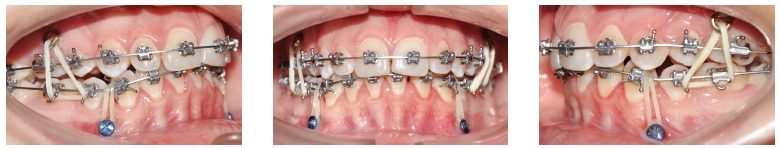
Post-surgical occlusion.

A month after condylectomy, an orthognathic jaw impaction and asymmetric mandibular setback surgery was performed with the SFA. It was decided to leave zygomatic plates (Y-shaped plate, Zimmer Biomet) for vertical control of posterior teeth using ¼-in 4.5 oz intermaxillary elastics (Ortho Organizers Inc.) from the zygomatic plate to the lower posterior teeth, and 1.6 x 6 mm miniscrews (Royal Brand) between canines and lower bicuspids, for vertical control of the anterior teeth using 3/16-in 4.5 oz intermaxillary elastics (Ortho Organizers Inc.) from the miniscrews to canines and also both systems could assist with the postoperative sagittal control (Fig 5). The surgeon removed the lower second molars buccal tubes during the surgical procedure in order to facilitate the lower third molars extraction.

After 10 days, orthodontic controls were made every two weeks during 4 months. This stage was completed with orofacial physiotherapy to help the lymphatic drainage, neuromuscular rehabilitation and neurosensory recovery. 

The sequence used was: 0.014-in and 0.018-in Sentalloy archwires (Dentsply GAC), followed by 0.020 x 0.020-in BioForce Archwires (Dentsply GAC), 0.019 x 0.025-in Stainless Steel Archwires (Dentsply GAC) and 0.019 x 0.025-in Braided upper an lower archwires (Dentsply GAC).

The active phase of treatment lasted 14 months and fixed appliances were removed to start the retention stage.

## TREATMENT RESULTS

**Figure 6:**
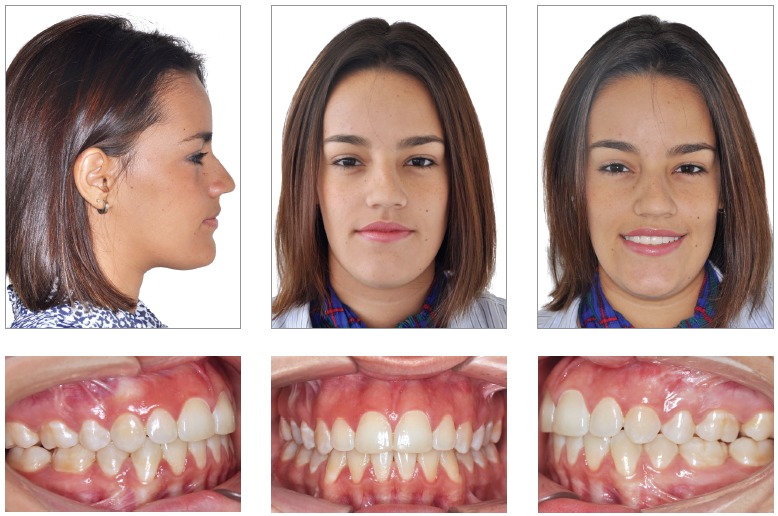
Final photographs.

**Figure 7:**
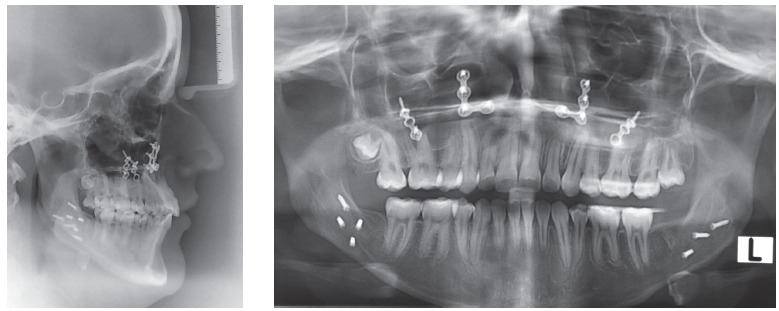
Final radiographs.

**Figure 8:**
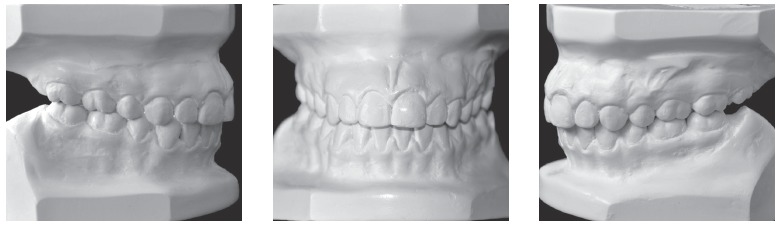
Final dental casts.

Post-treatment records confirmed that treatment was able to optimize the patient’s facial and dental esthetics, showing a considerable enhancement of the profile, correction of the Class III skeletal pattern and mandibular deviation, decreased lower facial height, improvement of the overjet and overbite, and alignment of the dental arches into a functional Class I occlusion (Figs 6, 7 and 8, Table 1).

**Figure 9:**
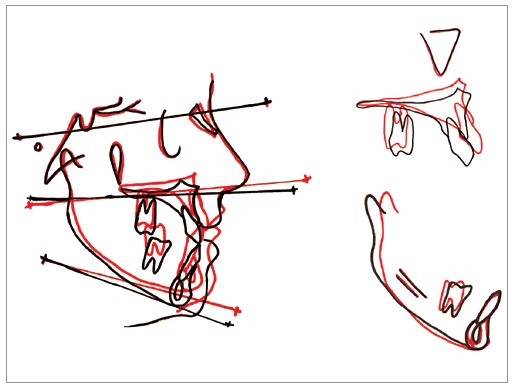
Superimposition: Initial and final cephalograms.

The superimposition showed decreased lower facial height and mandibular effective length, with counterclockwise rotation of mandible and maxilla (Fig 9).

**Figure 10:**
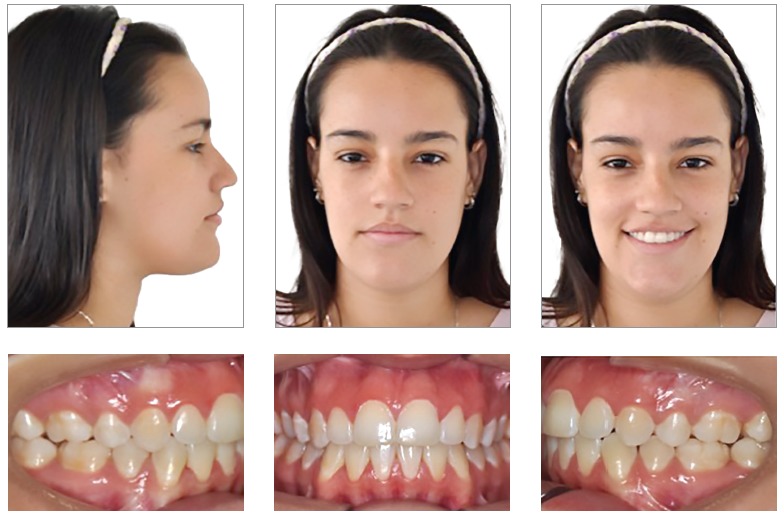
Photographs after 24 months in retention.

After 24 months in retention the results remained stable (Fig 10).

## DISCUSSION

Laterally deviated mandibles have many possible causes. The differential diagnosis of the laterally deviated mandible has some more common causes that include muscular torticollis, previous subcondylar or condylar fracture, condylar hyperplasia, juvenile condylar arthritis, hemifacial microsomia, deformational posterior plagiocephaly and unilateral coronal craniosynostosis. These causes may be grouped into two categories; however, there is some overlap. First, alteration of the cranial base may result in a laterally deviated mandible caused by the malpositioned glenoid fossa. This cranial base alteration group includes muscular torticollis, unilateral coronal craniosynostosis and deformational plagiocephaly. Second, condylar abnormality may result in a laterally deviated mandible from a hypoplasia or hyperplasia growth disturbance at the condylar site. This condylar abnormality group includes condylar fractures, condylar hyperplasia, hemifacial microsomia, and juvenile condylar arthritis. Proper diagnosis and subsequent treatment of the underlying abnormality of the deviated mandible ensure the patient of the appropriate orthognathic reconstruction and decrease the likelihood of skeletal relapse after surgery.^23^

Hyperplasia is defined as the growth of the number of cells present in a certain tissue with no increase in their size. When this affects the mandibular condylar head, it is called CH and is characterized by alterations in both the condylar and facial morphology.^10^

The histological report of this patient showed significant extensions of cartilage that exceeded the osteochondral junction, and increase in the thickness of the layers of the condyle, these characteristics were consistent with active condylar hyperplasia. Classifications have been developed in an attempt to standardize the concept of CH and its treatment,^24^ establishing a series of parameters associated with its clinical presentation and the conditions of diagnosis and treatment. The classification proposed by Slootweg and Müller^25^ includes limited histological aspects within the parameters; they established that different degrees of invasion of fibrocartilage islands are associated with the degree of aggressiveness of CH, classifying this from level 1 to 4. Saridin et al^26^ recently performed a histopathological study of unilateral condylar hyperplasia (UCH) using the classification proposed by Slootweg and Muüller. They reported that the identification of the four condylar layers at the histological level using hematoxylin-eosin is complex, and only two layers were used (the four initial ones were condensed) in order to identify them with greater clarity. The patterns described by Slootweg and Müller,^25^ Saridin et al,^26^ Eslami et al^2^ and Villanueva-Alcojol et al^6^ have not varied significantly in their histological descriptions. 

It is very important to establish a proper diagnosis of UCH to determine opportune treatment protocol that allows detain the progression of the disease and correct the aesthetic and functional consequences it produces.^27^ ^,^ ^28^ Also diagnosis and early intervention, prevent the establishment of facial asymmetry, as this is considered the main cause of postnatal facial asymmetries.^7^ The gold standard for diagnosis of UCH is correlating the clinical findings with the bone scan;^29^ once established, the next step is to determine whether this is an active or passive status, by means of a SPECT,^30^ for establishing whether cellular uptake osteoblast on suspicious condyle.^31^ A recent meta-analysis compared the sensitivity (true positive) and specificity (true negative) of a planar bone scan and the SPECT scanning technique in the diagnosis of UCH^32^ The sensitivity of the single photon emission computed tomography scan (0.91) was higher than that for the planar bone scan (0.70), whereas the specificity for the two techniques was similar (0.9). 

When the active state of the UCH is checked, the surgical protocol varies according to the patient’s age and asymmetry severity, but generally the treatment options proposed to date recommended condyle surgery and surgical correction of sequelae by orthognathic surgery, as the therapeutic approach of choice.^8^ ^,^ ^33^ ^,^ ^34^

In the present case the active state of UCH in the patient necessarily demanded a high condylectomy, but additionally, its consequences required an additional therapeutic approach as orthognathic surgery that could be done after an orthodontic preparation or, as it was performed, with SFA. 

Currently the most used concept of orthodontic treatment combined with orthognathic surgery is the three-phase approach, which consists of: 1) pre-surgery orthodontics for dental alignment, incisor decompensation and arch coordination, 2) orthognathic surgery with splints and rigid fixation to correct the skeletal discrepancies, and 3) post-surgery orthodontics to set in the occlusion.^35^ ^-^ ^43^ Even though these procedures usually have a positive outcome,^44^ disadvantages such as longer treatment time, deterioration of aesthetics and function are present during the pre-surgery orthodontic phase.^45^

In the classical approach, pre-surgery orthodontics usually takes up from 15 to 17 months,^46^ ^,^ ^47^ or even 24 months.^48^ Post-surgery orthodontics lasts between 7 and 12 months.^48^ In contrast, if orthognathic surgery is performed before the orthodontic treatment, total treatment time can be significantly reduced, and it would last even less than the average time it takes only to complete pre-surgery orthodontics.^46^ ^,^ ^47^ ^,^ ^49^

In this specific case, it was decided to do first the condylectomy and in second place the orthognathic surgery, having in mind that if the condylectomy and orthognathic surgery are done simultaneously, the intra-articular, skeletal, muscular and functional relationship are altered at the same time. Also, after condylectomy the condyle dramatically changes its position and affects its relationship with the joint, therefore the type and magnitude of orthognathic surgery could change.^50^

In this case report with the combination of proper diagnosis, excellent communication with the maxillofacial surgeon during the planning of surgical treatment composed by condylectomy and orthognatic surgery with SFA, and post-surgical orthodontics using temporary anchorage devices, it was possible to solve the aesthetic and functional problem of this patient with UCH.

## CONCLUSIONS

The relationship between a correct diagnosis, the importance of the timing of intervention and proper biomechanics showed aesthetic and functional results in this case of asymmetry.

With the condylectomy and SFA the results were successful in this case report, exhibiting aesthetic changes from baseline and appropriate treatment time.

## References

[1] Nitzan DW, Katsnelson A, Bermanis I, Brin I, Casap N (2008). The clinical characteristics of condylar hyperplasia experience with 61 patients. J Oral Maxillofac Surg.

[2] Eslami B, Behnia H, Javadi H, Khiabani KS, Saffar AS (2003). Histopathologic comparison of normal and hyperplastic condyles. Oral Surg Oral Med Oral Pathol Oral Radiol Endod.

[3] Raijmakers PG, Karssemakers LH, Tuinzing DB (2012). Female predominance and effect of gender on unilateral condylar hyperplasia a review and meta-analysis. J Oral Maxillofac Surg.

[4] López DF, Corral C (2015). Hiperplasia condilar características, manifestaciones, diagnóstico y tratamiento. Rev Fac Odontol Univ Antioq.

[5] Obwegeser HL, Makek MS (1986). Hemimandibular hyperplasia-hemimandibular elongation. J Maxillofac Surg.

[6] Villanueva-Alcojol L, Monje F, González-García R (2011). Hyperplasia of the mandibular condyle clinical, histopathologic, and treatment considerations in a series of 36 patients. J Oral Maxillofac Surg.

[7] Wen B, Shen Y, Wang CY (2014). Clinical value of 99Tcm-MDP SPECT bone scintigraphy in the diagnosis of unilateral condylar hyperplasia. Sci World J.

[8] Hodder SC, Rees JI, Oliver TB, Facey PE, Sugar AW (2000). SPECT bone scintigraphy in the diagnosis and management of mandibular condylar hyperplasia. Br J Oral Maxillofac Surg.

[9] Alyamani A, Abuzinada S (2012). Management of patients with condylar hyperplasia a diverse experience with 18 patients. Ann Maxillofac Surg.

[10] Olate S, Duque H, Rodriguez J, Alister J, Barbosa A, Moraes M (2013). Mandible condylar hyperplasia a review of diagnosis and treatment protocol. Int J Clin Exp Med.

[11] Muñoz MF, Monje F, Goizueta C, Rodríguez-Campo F (1999). Active condylar hyperplasia treated by high condilectomy report of case. J Oral Maxillofac Surg.

[12] Angiero F, Farronato G, Benedicenti S, Vinci R, Farronato D, Magistro S (2009). Mandibular condylar hyperplasia clinical, histopathological, and treatment considerations. Cranio.

[13] Villegas C, Uribe F, Sugawara J, Nanda R (2010). Expedited correction of significant dentofacial asymmetry using a "surgery first" approach. J Clin Orthod.

[14] Villegas C, Janakiraman N, Uribe F, Nanda R (2012). Rotation of the maxilla mandibular complex to enhance esthetics using a "surgery first" approach. J Clin Orthod.

[15] Hong KJ, Lee JG (1999). 2 phase treatment without preoperative orthodontics in skeletal Class III malocclusion. Korean J Oral Maxillofac Surg.

[16] Nagasaka H, Sugawara J, Kawamura H, Nanda R (2009). "Surgery first" skeletal Class III correction using the skeletal anchorage system. J Clin Orthod.

[17] Aristizábal JF, Martínez Smit R, Villegas C (2015). The "surgery first" approach with passive self-ligating brackets for expedited treatment of skeletal Class III malocclusion. J Clin Orthod.

[18] Aristizábal JF (2014). Accelerated orthodontics and express transit orthodontics (ETO)(r), a contemporary concept of high efficiency. CES Odontol.

[19] Liou EJ, Chen PH, Wang YC, Yu CC, Huang CS, Chen YR (2011). Surgery-first accelerated orthognathic surgery postoperative rapid orthodontic tooth movement. J Oral Maxillofac Surg.

[20] Frost HM (1989). The biology of fracture healing An overview for clinicians. Part I. Clin Orthop Relat Res.

[21] Frost HM (1989). The biology of fracture healing An overview for clinicians. Part II. Clin Orthop Relat Res.

[22] López BDF, Corral SCM (2016). Comparison of planar bone scintigraphy and single photon emission computed tomography for diagnosis of active condylar hyperplasia. J Craniomaxillofac Surg.

[23] Kawamoto HK, Kim SS, Jarrahy R, Bradley JP (2009). Differential diagnosis of the idiopathic laterally deviated mandible. Plast Reconstr Surg.

[24] Wolford LM, Movahed R, Perez DE (2014). A classification system for conditions causing condylar hyperplasia. J Oral Maxillofac Surg.

[25] Slootweg PJ, Müller H (1986). Condylar hyperplasia A clinico-pathological analysis of 22 cases. J Maxillofac Surg.

[26] Saridin CP, Raijmakers PG, Slootweg PJ, Tuinzing DB, Becking AG, van der Waal I (2010). Unilateral condylar hyperactivity a histopathologic analysis of 47 patients. J Oral Maxillofac Surg.

[27] Diaz PM, Garcia RG, Gias LN, Aguirre-Jaime A, Pérez JS, de la Plata MM (2010). Time used for orthodontic surgical treatment of dentofacial deformities in white patients. J Oral Maxillofac Surg.

[28] Luther F, Morris DO, Karnezi K (2007). Orthodontic treatment following orthognathic surgery how long does it take and why? A retrospective study. J Oral Maxillofac Surg.

[29] Saridin CP, Raijmakers P, Becking AG (2007). Quantitative analysis of planar bone scintigraphy in patients with unilateral condylar hyperplasia. Oral Surg Oral Med Oral Pathol Oral Radiol Endod.

[30] Elbaz J, Wiss A, Raoul G, Leroy X, Hossein-Foucher C, Ferri J (2014). Condylar hyperplasia correlation between clinical, radiological, scintigraphic, and histologic features. J Craniofac Surg.

[31] Wolford LM, Morales-Ryan CA, García-Morales P, Peréz D (2009). Surgical management of mandibular condylar hyperplasia type 1. Proc (Bayl Univ Med Cent).

[32] Saridin CP, Raijmakers PG, Tuinzing DB, Becking AG (2011). Bone scintigraphy as a diagnostic method in unilateral hyperactivity of the mandibular condyles a review and meta-analysis of the literature. Int J Oral Maxillofac Surg.

[33] Gn S, Sharma ML, Jk DR, Goel S, Srivastava S (2015). Facial asymmetry in young adults with condylar hyperplasia-unusual changes in the facial bones. J Clin Diagn Res.

[34] Front D, Israel O, Jerushalmi J, Frenkel A, Iosilevsky G, Feinsod M (1989). Quantitative bone scintigraphy using SPECT. J Nucl Med.

[35] Behrman SJ, Behrman DA (1988). Oral surgeons' considerations in surgical orthodontic treatment. Dent Clin North Am.

[36] Troy BA, Shanker S, Fields HW, Vig K, Johnston W (2009). Comparison of incisor inclination in patients with Class III malocclusion treated with orthognathic surgery or orthodontic camouflage. Am J Orthod Dentofacial Orthop.

[37] Bell WH, Creekmore TD (1973). Surgical-orthodontic correction of mandibular prognathism. Am J Orthod.

[38] Worms FW, Isaacson RJ, Speidel TM (1976). Surgical orthodontic treatment planning profile analysis and mandibular surgery. Angle Orthod.

[39] Vig KD, Ellis 3rd E (1990). Diagnosis and treatment planning for the surgical-orthodontic patient. Dent Clin North Am.

[40] Verdier M, Ghadanfar A, Coutant A (1994). Planning of pre- and post-surgical orthodontic alignments. Rev Stomatol Chir Maxillofac.

[41] Proffit WR, Miguel JA (1995). The duration and sequencing of surgical-orthodontic treatment. Int J Adult Orthodon Orthognath Surg.

[42] Bousaba S, Delatte M, Barbarin V, Faes J, De Clerck H (2002). Pre- and post-surgical orthodontic objectives and orthodontic preparation. Rev Belge Med Dent (1984).

[43] Proffit WR, White RP, Proffit WR, White RP, Sarver DM (2003). Combining surgery and orthodontics: who does what, when?. Contemporary treatment of dentofacial deformity.

[44] Sabri R (2006). Orthodontic objectives in orthognathic surgery: state of the art today. World J Orthod.

[45] Proffit WR, Turvey TA, Phillips C (2007). The hierarchy of stability and predictability in orthognathic surgery with rigid fixation an update and extension. Head Face Med.

[46] Cunningham SJ, Hunt NP, Feinmann C (1996). Perceptions of outcome following orthognathic surgery. Br J Oral Maxillofac Surg.

[47] Nurminen L, Pietilä T, Vinkka-Puhakka H (1999). Motivation for and satisfaction with orthodontic-surgical treatment a retrospective study of 28 patients. Eur J Orthod.

[48] Luther F, Morris DO, Hart C (2003). Orthodontic preparation for orthognathic surgery How long does it take and why? A retrospective study. Br J Oral Maxillofac Surg.

[49] Dowling PA, Espeland L, Krogstad O, Stenvik A, Kelly A (1999). Duration of orthodontic treatment involving orthognathic surgery. Int J Adult Orthodon Orthognath Surg.

[50] El mozen LA, Meng QG, Li YJ, Long X, Chen GX (2015). Condylar and occlusal changes after high condylectomy and orthodontic treatment for condylar hyperplasia. J Huazhong Univ Sci Technolog Med Sci.

